# The use of therapeutic drug monitoring to highlight an over-looked drug-drug interaction leading to imatinib treatment failure

**DOI:** 10.1007/s40199-023-00465-z

**Published:** 2023-06-15

**Authors:** Sara Gagno, Angela Buonadonna, Chiara Dalle Fratte, Michela Guardascione, Martina Zanchetta, Bianca Posocco, Marco Orleni, Giovanni Canil, Rossana Roncato, Erika Cecchin, Giuseppe Toffoli

**Affiliations:** 1 Experimental and Clinical Pharmacology Unit, Centro di Riferimento Oncologico di Aviano (CRO), IRCCS, Aviano, Italy; 2 Department of Medical Oncology, Centro di Riferimento Oncologico di Aviano (CRO), IRCCS, Aviano, Italy

**Keywords:** Therapeutic drug monitoring, Imatinib, Carbamazepine, Drug-drug interaction

## Abstract

**Background:**

Chronic oral anticancer therapies, are increasingly prescribed and present new challenges including the enhanced risk of overlooked drug-drug interactions (DDIs). Lengthy treatments and patients’ management by different professionals can lead to serious prescribing errors that therapeutic drug monitoring (TDM) can help identifying thus allowing a more effective and safer treatment of patients with polypharmacy.

**Objectives:**

This report aims to exemplify how an intensified pharmacological approach could help in the clinical monitoring of patients on chronic treatments.

**Methods:**

A patient with gastrointestinal stromal tumor was referred to our clinical pharmacology service due to tumor progression while on imatinib therapy. The investigation was based on TDM, pharmacogenetics, DDI evaluation and Circulating tumor DNA (ctDNA) analysis. The patient underwent repeated blood samplings to measure imatinib and norimatinib plasma concentrations through a validated LC-MS/MS method. Polymorphisms affecting genes involved in imatinib metabolism and transport were investigated using SNPline PCR Genotyping System. Drug-drug interactions were evaluated though Lexicomp. ctDNA analysis was performed on MiSeq platform.

**Results:**

TDM analysis revealed that the patient was underexposed to imatinib (C_min_ = 406 ng/mL; target C_min_ = 1100 ng/mL). Subsequent DDI analysis highlighted a dangerous interaction with carbamazepine, via CYP3A4 and P-gp strong induction, omitted at the time of imatinib treatment start. No relevant pharmacogenetic variants were identified and appropriate compliance to treatment was ascertained. ctDNA monitoring was performed to assess potential tumor-related resistance to imatinib. Carbamazepine was cautiously switched to a non-interacting antiepileptic drug, restoting IMA plasma concentration (i.e. C_min_ = 4298 ng/mL). The progression of the disease, which in turn led to the patient’s death, was also witnessed by an increasing fraction of ctDNA in plasma.

**Conclusion:**

The active pharmacological monitoring allowed the identification of a dangerous previously over-looked DDI leading to IMA under-exposure. The switch to a different antiepileptic treatment, reversed the effect of DDI, restoring therapeutic IMA plasmatic concentrations.

**Graphical abstract:**

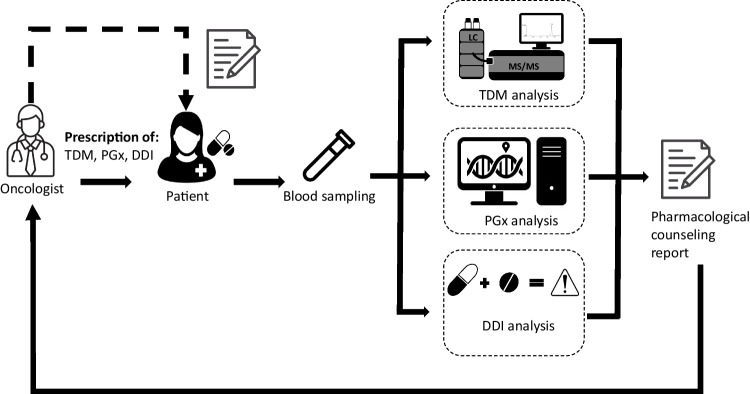

**Supplementary Information:**

The online version contains supplementary material available at 10.1007/s40199-023-00465-z.

## Introduction

Large interindividual variability in plasma exposure to oral anticancer drugs administered at a fixed dose has been reported, affecting the efficacy and toxicity of therapy [1]. Causes include the use of multiple drugs that place patients at risk for drug-drug interactions (DDIs) and pharmacogenetic profile that could influence individual metabolic capacity. Recent literature indicates that a large number of medication errors are associated with the use of oral anticancer drugs, including imatinib (IMA), due to poor attention to patient polypharmacy, resulting in additional toxicity burden for patients and variable exposure [1, 2].

IMA is a potent tyrosine kinase inhibitor (TKI) indicated for the treatment of gastrointestinal stromal tumors (GIST) and chronic myeloid leukemia. Its biotransformation occurs mainly in the liver, where it is metabolized primarily by CYP3A4. Its main N-desmethyl metabolite, norimatinib (NOR), has similar biological activity and accounts for approximately 20-25% of drug levels at steady state (NOR /IMA ratio) [3]. IMA is also a substrate of P-glycoprotein (Pgp - ABCB1) which mediates its re-extrusion into the intestinal lumen and elimination from cancer cells. In patients with GIST, a IMA threshold C_min_ (minimum concentration before the next drug dose) of 1100 ng/mL has been recommended as a target concentration for efficacy [4]. Suboptimal plasma levels of IMA have been associated with worse outcome in the past [3, 5].

Carbamazepine is an enzyme-inducing antiepileptic drug (EIAED) that acts as a potent inducer of CYP3A4 and PgP. Co-administration with IMA accelerates its metabolism and alters its intestinal absorption, reducing exposure. A special warning in IMA summary of product characteristics recommends avoiding concomitant administration of IMA with CYP3A4 inhibitors, inducers, or substrates, but does not provide advice on the management of concomitant administration in patients in whom the use of these drugs is required [6]. Initiating IMA treatment in a patient already taking carbamazepine without noticing this DDI could lead to dangerous long-term patient undertreatment.

Here, we describe how integrating intensified pharmacologic care into the clinical management of cancer patients can be useful in highlighting situations that require treatment adjustment. In particular, we report a patient treated with IMA (GIST) in whom the combined approach of therapeutic drug monitoring (TDM), pharmacogenetics, and DDI analysis revealed the presence of a DDI with preexisting carbamazepine treatment. Although the interaction between imatinib and carbamazepine is well known and concomitant administration is discouraged, it was initially overlooked, leading to several years of patient under treatment for GIST. In addition, a circulating tumor free DNA (ctDNA) monitoring strategy was used as a complementary tool to confirm disease progression.

## Methods

### Patient’s samplings

Patient’s blood samplings were obtained after he signed a written informed consent. A 2.7 mL K-EDTA tube of blood was collected at each sampling and immediately centrifuged at 2450 g for 10 min at 4 °C. The obtained plasma was stored at −80 °C until analysis. Sampling for ctDNA were collected on three 4.9 mL K-EDTA tubes, which were double centrifuged within 1 hour from drawn (first centrifugation at 1600 g for 10 min without brake; the isolated plasma was centrifuged again at 3400 g for 10 min without brake). The buffy coat was collected for pharmacogenetics analysis and stored at −80 °C until DNA extraction. IMA and NOR plasma concentrations were determined at steady state at the C_min_, that is at least after 5 days of continuous treatment at the same dose and after 24 hours from last drug intake in case of treatment at 400 mg once daily (OD) and after 12 hours in case of treatment at 400 mg twice daily (BID).

### LC/MS-MS quantification of IMA and NOR plasma concentrations

Patient’s samples were quantified using a LC-MS/MS system composed by a Prominence UFLC XR (Shimadzu, Tokyo, Japan) coupled with an API 4000 QTrap mass spectrometer (SCIEX, Massachusetts, USA) using an in-house method validated according to EMA and FDA guidelines [7, 8]. Briefly, IMA and NOR were extracted from plasma with a simple protein precipitation. The analytes were separated on a Synergi Fusion RP C18 chromatographic column 4 μm, 50 × 2.0 mm coupled with a C18 precolumn provided by Phenomenex (Torrance, CA, USA). The concentrations of the calibration curve (30-7500 ng/mL) cover those expected to be found in patients’ plasma. The intra- and inter-day precision resulted ≤4.7% and ≤ 7.1% for IMA and ≤ 6.4% and ≤ 7.2% for NOR, respectively. The intra- and inter-day accuracy for IMA was between 92.7-104.7% and 99.5-103.8%, respectively, while for NOR ranged from 95.2% to 104.7% and from 98.0% to106.3%. The Limit of Detection (LOD) resulted 2.1 and 0.9 ng/mL, for IMA and NOR, respectively. The Lower Limit Of Quantification (LLOQ) was fixed at 30 ng/mL for IMA and 6 ng/mL for NOR. No interference was highlighted at analytes’ retention times. Further details on the LC-MS/MS method are available upon request.

### Germ-line DNA analysis

The patient was genotyped for polymorphisms in relevant IMA metabolism (*CYP3A4*, *CYP3A5*) and transporters (*ABCB1*, and *ABCG2*) genes (Table 1). The pharmacogenetic analysis was performed by SNPline PCR Genotyping System platform employing Kompetitive allele-specific PCR (KASP) assays (LGC Genomics, Hoddesdon, UK) according to manufacturer’s instructions (Biosearch Technologies). The tri-allelic discrimination of *ABCB1* 2677G > T/A was assessed using Pyrosequencing (PyroMark Q48; Qiagen, Hilden, Germany). Positive and negative control samples were included in each analysis. Primer sequences and genotyping details are available upon request.

**Table 1:** Patient’s pharmacogenetic profile

Gene	Polymorphism	Rs_ID	Base change	Genotype
CYP3A4	CYP3A4*1B	rs2740574	C > A,G,T	AA
CYP3A4	CYP3A4*3	rs4986910	T > C	TT
CYP3A4	CYP3A4*20	rs67666821	delT/dupT	delT/delT
CYP3A4	CYP3A4*22	rs35599367	C > T	CC
CYP3A5	CYP3A5*3	rs776746	A > G	GG
CYP3A5	CYP3A5*6	rs10264272	C > T	CC
CYP3A5	CYP3A5*7	rs41303343	- > A	−/−
ABCB1	ABCB1 c.1236C > T (p.G412=)	rs1128503	C > T	CC
ABCB1	ABCB1 c.3435C > T (p.I1145=)	rs1045642	C > T	CC
ABCB1	ABCB1 c.2677G > T/A (p.S893A)	rs2032582	G > T,A	GG

### ctDNA analysis

Total cell-free DNA (cfDNA) was extracted from plasma using the QIAamp MinElute ccfDNA Kit (Qiagen) and quantified with Quantus Fluorometer (Promega). Fragment size distribution was assessed by High Sensitivity Tapestation (Agilent Technologies). Genomic libraries were prepared using a customised QiaSeq Panel DNA (Qiagen), encompassing the coding sequence of 13 GIST-related genes (Supplementary Table 1). Targeted libraries were sequenced in a MiSeq platform (Illumina). Bioinformatic analysis was performed on a workstation with a 30 core Intel Core i7 running Centos 7.5. Variants were called using smCounter v2 with default parameters [9].

## Results

### Case presentation

A 63-year-old man was referred to our clinical pharmacology service due to disease progression on IMA therapy. The patient was diagnosed with abdominal GIST by biopsy of liver metastases. Immunohistochemical evaluation of the biopsy specimen revealed c- KIT expression, but no data on the molecular features of the tumour were available. At diagnosis, first-line treatment with IMA 400 mg OD (Gleevec, Novartis) was started, administered continuously, and achieved a partial response as the best clinical response in almost 6 years. When CT showed evidence of progressive disease in the abdominal lesion, pharmacologic counselling was requested.

### Therapeutic drug monitoring and drug-drug interaction analysis

Blood was drawn from the patient after signing a written informed consent form, and the plasma concentration of the drug was determined using an internally validated LC-MS method (more details can be found in [10]). TDM analysis revealed a IMA C_min_ of 406 ng/mL and NOR C_min_ of 213 ng/mL. The possible causes for this low exposure were investigated. Treatment adherence was considered adequate based on patient reports. To investigate DDIs of potential clinical significance, the patient’s medical treatments were reviewed. This revealed that the patient also had epilepsy, which had been controlled with carbamazepine 200 mg daily for nearly 30 years. In addition, he was taking pantoprazole for gastroesophageal reflux and ramipril/amlodipine to control hypertension. Potential DDIs were investigated using Lexicomp and the summary of characteristics of each co-administered drug (EMA).

Although pantoprazole can increase the exposure of IMA and amlodipine concentration is increased by IMA [11], the most significant DDI was identified between IMA and carbamazepine, which is known to be a potent inducer of CYP3A4 and P-gp [12, 13]. The observed NOR /IMA ratio in the patient sample was 52.5% (normally in the range of 20-25%), confirming an induction of metabolism. Meanwhile, as a consequence of the patient’s disease progression, the dose of IMA was increased to 400 mg twice daily (BID) and plasma concentration was re-monitored once it reached the new steady state to determine if this dose increase would have been sufficient to raise the plasma concentration of IMA to the target value of 1100 ng/mL.

The first sampling after dose escalation showed an IMA Cmin of 892 ng/mL, with a NOR /IMA ratio of 51.9%. Although the concentration of IMA increased slightly, it was still below the plasma levels associated with efficacy, and the NOR /IMA ratio was unchanged. Because no other options for dose escalation were feasible, the patient was informed and asked to talk with his neurologist about switching to a non-EIAED drug. Switching to another drug would have been beneficial for second-line therapy of GIST because sunitinib is also a substrate of CYP3A4 and thus a victim of CYP3A4 induction by carbamazepine.

**Fig. 1:**
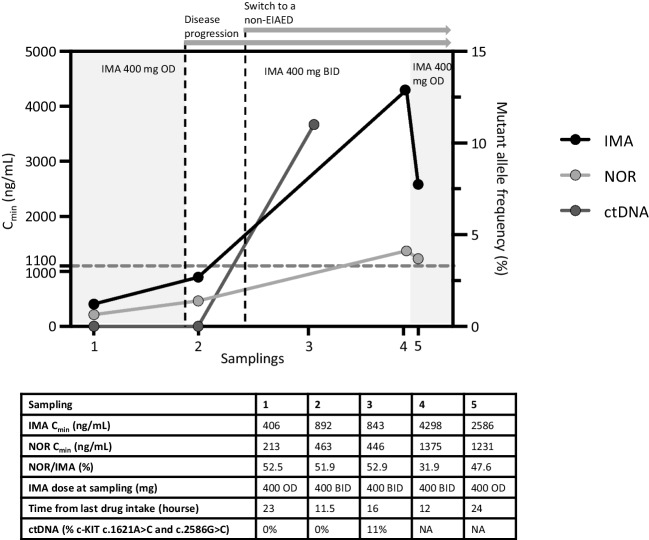
Graphical representation of patient’s clinical history time-line with variation of IMA, NOR and ctDNA concentrations levels. Dashed grey line represents the C_min_ threshold of 1100 ng/mL recommended for GIST patients. Grey backgrounds represent treatment at 400 mg OD, while white background identifies IMA treatment at 400 mg BID. Dashed black vertical lines indicate disease progression and switch to lacosamide, respectively. Abbreviations: IMA: imatinib, NOR: norimatinib, ctDNA: circulating tumor DNA, OD: once daily, BID: bis in die (twice daily), non-EIAED: non enzyme inducing antiepileptic drug, GIST: gastrointestinal stromal tumors

The patient’s neurologist established a planned switch over four months in which carbamazepine was de-escalated and lacosamide, a non-EIAED, was gradually increased. Two weeks before completion of the switch (i.e., while the patient was receiving 50 mg carbamazepine and 100 mg lacosamide), the IMA level was measured again, revealing a markedly increased IMA concentration of 4298 ng/mL (NOR =1375 ng/mL) and a partial normalization of the NOR /IMA ratio (31.9%), synonymous with reversal of the effect of carbamazepine. Because of this high concentration, the oncologist decided to decrease the dose of IMA to 400 mg OD (to reduce the risk of adverse toxicity to the patient) and to reassess the concentration of IMA after 1 month, an appropriate time period since the reversal of carbamazepine induction can be considered complete within 2 weeks after cessation of carbamazepine therapy [14]. The concentration of IMA was 2586 ng/mL, which was lower than the previous determination but still within the therapeutic range (Fig. 1) [15].

### Pharmacogenetic analyses

The analysis included the study of polymorphisms affecting the main IMA metabolizing enzymes (*CYP3A4* and *CYP3A5)* and transcellular transporters (*ABCB1* and *ABCG2)*. No significant pharmacogenetic markers were identified that could potentially affect patients’ exposure to the drug (Table 1).

### Circulating tumor DNA analysis

To elucidate potential alternative causes of tumor resistance to IMA, ctDNA was studied during treatment. Specifically, a panel of GIST-related genes in patient ctDNA was analysed (Supplementary Table 1) to investigate somatic changes potentially responsible for imatinib resistance. Two *c-KIT* mutations, c.1621A > C and c.2586G > C (Supplementary Table 2), were detected simultaneously during the course of IMA 400 mg BID, both with an allele frequency of 11% (Fig. 1). Although not associated with the development of IMA resistance, the presence of *c-KIT* mutations, tracking tumor DNA in plasma, further supports the clinical diagnosis of disease progression.

### Case outcome

The applied TDM approach combined with pharmacogenetic analysis and comprehensive review of the DDI identified a case of severe imatinib underexposure after several years of concomitant treatment with the interacting drug carbamazepine. With the support of the neurologist, carbamazepine was gradually switched to lacosamide, a non-EIAED drug. Plasma levels of imatinib rose back above the efficacy threshold. Disease progression was confirmed by an increase in plasma ctDNA levels. Unfortunately, the patient’s disease progressed steadily during this time and resulted in death.

## Discussion

This case report provides evidence of the potential clinical effectiveness of integrated drug monitoring to support the management of patients chronically treated with a fixed-dose oral anticancer drug such as imatinib. We described the case of a patient with GIST, who was found to be severely underexposed to the drug after IMA treatment failure and the cause was found to be a previously overlooked DDI with carbamazepine.

As described in detail previously, IMA can interact with a wide range of CYP3A4 and P-gp substrates [11, 16, 17], and the interaction between carbamazepine and IMA is well established. The inducing effect of carbamazepine on CYP3A4 and possibly also on P-gp likely led to a decrease in circulating levels of IMA and thus to treatment inefficacy.

Pursche and colleagues examined the effect of various EIAEDs on the pharmacokinetics of IMA in a cohort of 224 glioblastoma patients. They reported an average trough IMA level of 473 ng/mL in patients taking carbamazepine compared with 1404 ng/mL in patients not taking an antiepileptic drug, and consistent with our observation, the NOR /IMA ratio in patients taking carbamazepine was approximately 50.7% [18].

As in the case reported here, it has been described that about 30% of cancer patients are exposed to potentially dangerous drug combinations [19] and that significant DDIs are more common in patients taking oral anticancer drugs than in patients treated with injectable agents [20, 21]. Improved pharmacologic monitoring that allows early detection of otherwise neglected dangerous interactions has been shown to improve patient safety and quality of life [2]. In addition, interrogation of tumor-derived DNA by liquid biopsy can be a useful tool for monitoring tumor progression in GIST [22]. Although this approach is already recommended in international guidelines for monitoring other solid tumors such as colorectal or lung cancer, it is not yet a routine practice in GIST patients. Although the two cKIT mutations detected in this case report have never been associated with IMA resistance and are unlikely to lead to disease progression, a liquid biopsy approach could be considered to noninvasively detect the onset of disease progression even in GIST.

## Conclusion

Chronic daily oral administration of cancer therapies, while offering undeniable advantages in terms of flexibility and convenience of (self-)administration, also presents new challenges, including potentially overlooked DID issues. We reported here evidence of a real-world, clinically relevant case of DDI between IMA and carbamazepine using TDM. The lesson from this experience is that early pharmacological counselling based on TDM, pharmacogenetics, DDI analysis, and ctDNA monitoring can be a useful tool to identify potentially dangerous situations that need prompt treatment with therapeutic adjustments.

### Supplementary Information

ESM 1 (DOCX 15 kb)

## Data Availability

All the relevant data are reported within the paper. For additional details, data are available on request to the authors.

## References

[1] Dürr P, Schlichtig K, Kelz C, Deutsch B, Maas R, Eckart MJ (2021). The randomized AMBORA trial: impact of pharmacological/pharmaceutical care on medication safety and patient-reported outcomes during treatment with new Oral anticancer agents. J Clin Oncol.

[2] Leenhardt F, Alexandre M, Guiu S, Pouderoux S, Beaujouin M, Lossaint G, et al. Impact of pharmacist consultation at clinical trial inclusion: an effective way to reduce drug-drug interactions with oral targeted therapy. Cancer Chemother Pharmacol. 2021;88:723–9. 10.1007/s00280-021-04331-0 34286354

[3] Demetri GD, Wang Y, Wehrle E, Racine A, Nikolova Z, Blanke CD, et al. Imatinib plasma levels are correlated with clinical benefit in patients with unresectable/metastatic gastrointestinal stromal tumors. J Clin Oncol. 2009;27:3141–7. 10.1200/JCO.2008.20.4818 19451435

[4] Clarke WA, Chatelut E, Fotoohi AK, Larson RA, Martin JH, Mathijssen RHJ (2021). Therapeutic drug monitoring in oncology: International Association of Therapeutic Drug Monitoring and Clinical Toxicology consensus guidelines for imatinib therapy. Eur J Cancer.

[5] Bouchet S, Poulette S, Titier K, Moore N, Lassalle R, Abouelfath A (2016). Relationship between imatinib trough concentration and outcomes in the treatment of advanced gastrointestinal stromal tumours in a real-life setting. Eur J Cancer.

[6] EMA imatinib. Summary of product characteristics (Imatinib) [Internet]. Available from: https://www.ema.europa.eu/en/documents/product-information/glivec-epar-product-information_en.pdf. Accessed 1 May 2023.

[7] EMA. Guideline on bioanalytical method validation [Internet]. 2012. Available from: https://www.ema.europa.eu/en/documents/scientific-guideline/guideline-bioanalytical-method-validation_en.pdf. Accessed 1 May 2023.

[8] FDA. Bioanalytical Method Validation Guidance for Industry [Internet]. 2018. Available from: https://www.fda.gov/files/drugs/published/Bioanalytical-Method-Validation-Guidance-for-Industry.pdf. Accessed 1 May 2023.

[9] Xu C, Gu X, Padmanabhan R, Wu Z, Peng Q, DiCarlo J (2019). smCounter2: an accurate low-frequency variant caller for targeted sequencing data with unique molecular identifiers. Bioinformatics..

[10] Dalle Fratte C, Gagno S, Roncato R, Polesel J, Zanchetta M, Buzzo M, et al. CYP2D6 and CYP2C8 pharmacogenetics and pharmacological interactions to predict imatinib plasmatic exposure in GIST patients. Br J Clin Pharmacol. 2022; 10.1111/bcp.15551 36178950

[11] Haouala A, Widmer N, Duchosal MA, Montemurro M, Buclin T, Decosterd LA (2011). Drug interactions with the tyrosine kinase inhibitors imatinib, dasatinib, and nilotinib. Blood..

[12] FDA. Drug Development and Drug Interactions | Table of Substrates, Inhibitors and Inducers [Internet]. 2020. Available from: https://www.fda.gov/drugs/drug-interactions-labeling/drug-development-and-drug-interactions-table-substrates-inhibitors-and-inducers. Accessed 1 May 2023.

[13] Fuhr LM, Marok FZ, Hanke N, Selzer D, Lehr T (2021). Pharmacokinetics of the CYP3A4 and CYP2B6 inducer carbamazepine and its drug-drug interaction potential: a physiologically based pharmacokinetic modeling approach. Pharmaceutics..

[14] Punyawudho B, Cloyd JC, Leppik IE, Ramsay RE, Marino SE, Pennell PB (2009). Characterization of the time course of carbamazepine deinduction by an enzyme turnover model. Clin Pharmacokinet.

[15] Osorio S, Escudero-Vilaplana V, Gómez-Centurión I, González-Arias E, García-González X, Díez JL (2019). Inadequate response to imatinib treatment in chronic myeloid leukemia due to a drug interaction with phenytoin. J Oncol Pharm Pract.

[16] Elmeliegy M, Vourvahis M, Guo C, Wang DD (2020). Effect of P-glycoprotein (P-gp) inducers on exposure of P-gp substrates: review of clinical drug-drug interaction studies. Clin Pharmacokinet.

[17] van Leeuwen RWF, van Gelder T, Mathijssen RHJ, Jansman FGA (2014). Drug-drug interactions with tyrosine-kinase inhibitors: a clinical perspective. Lancet Oncol.

[18] Pursche S, Schleyer E, von Bonin M, Ehninger G, Said SM, Prondzinsky R (2008). Influence of enzyme-inducing antiepileptic drugs on trough level of imatinib in glioblastoma patients. Curr Clin Pharmacol.

[19] Riechelmann RP, Krzyzanowska MK (2019). Drug interactions and oncological outcomes: a hidden adversary. Ecancermedicalscience..

[20] Rogala BG, Charpentier MM, Nguyen MK, Landolf KM, Hamad L, Gaertner KM (2019). Oral anticancer therapy: Management of Drug Interactions. J Oncol Pract.

[21] Weingart SN, Brown E, Bach PB, Eng K, Johnson SA, Kuzel TM (2008). NCCN task force report: Oral chemotherapy. J Natl Compr Cancer Netw.

[22] Jilg S, Rassner M, Maier J, Waldeck S, Kehl V, Follo M (2019). Circulating cKIT and PDGFRA DNA indicates disease activity in gastrointestinal stromal tumor (GIST). Int J Cancer.

