# Supplementary material for: The use of therapeutic drug monitoring to highlight an over-looked drug-drug interaction leading to imatinib treatment failure
Source: Daru. 2023 Jun 15;31(2):267–72. doi: 10.1007/s40199-023-00465-z (PMC10624793; doi:10.1007/s40199-023-00465-z)
Supplement: Supplementary file 1 — (DOCX 15 kb) [file 40199_2023_465_MOESM1_ESM.docx]

**Article title:** Drug-drug interaction between imatinib and carbamazepine: a case report on the role of Therapeutic Drug Monitoring

**Journal name:** Clinical Pharmacokinetics

**Author names:** Gagno S*^1^, Buonadonna A^2^; Dalle Fratte C^1^; Guardascione M^2^; Zanchetta M.^1^, Posocco B^1^; Orleni M^1^; Canil G^1^, Roncato R.^1^, Cecchin E^1^; Toffoli G^1^.

* Author for correspondence:

Sara Gagno, PhD

Experimental and Clinical Pharmacology Unit, Centro di Riferimento Oncologico di Aviano (CRO), IRCCS

sgagno@cro.it

+39 0434 659783

**Supplementary Table 1.** List of genes selected for the targeted deep sequencing of codifying regions in the cfDNA of GIST patients. The gene name, the genomic coordinates, the transcript IS and the sequence ID (RefSeq) are displayed for each gene.

| Gene | Genome Position | Transcript ID | RefSeq |
| --- | --- | --- | --- |
| KIT | Chr4: 54,657,918-54,740,715 | ENST00000288135.6 | NM_000222.3 |
| PDGFRα | Chr4: 54,229,280-54,298,245 | ENST00000257290.10 | NM_006206.6 |
| MTOR | Chr1: 11,106,535-11,262,551 | ENST00000361445.9 | NM_004958.4 |
| BRAF | Chr7: 140,719,327-140,924,929 | ENST00000644969.2 | NM_001374258.1 |
| PIK3CA | Chr3: 179,148,114-179,240,093 | ENST00000263967.4 | NM_006218.4 |
| KRAS | Chr12: 25,205,246-25,250,936 | ENST00000256078.10 | NM_033360.4 |
| PTEN | Chr10: 87,863,625-87,971,930 | ENST00000371953.8 | NM_000314.8 |
| SRC | Chr20: 37,344,685-37,406,050 | ENST00000373578.7 | NM_198291.3 |
| STAT3 | Chr17: 42,313,324-42,388,482 | ENST00000264657.10 | NM_139276.3 |
| FIP1L1 | Chr4: 53,377,641-53,460,862 | ENST00000337488.11 | NM_030917.4 |
| MAX | Chr14: 65,006,174-65,102,695 | ENST00000358664.9 | NM_002382.5 |
| FGFR1 | Chr8: 38,400,215-38,468,834 | ENST00000447712.7 | NM_023110.3 |
| TP53 | Chr17: 7,661,779-7,687,538 | ENST00000269305.9 | NM_000546.6 |

**Supplementary Table 2.** c-KIT somatic mutations detected in the cfDNA by means of deep sequencing.

| Gene | ID | HGVS.c | HGVS.p | VAF | Depth (X) | UMI Depth (X) |
| --- | --- | --- | --- | --- | --- | --- |
| KIT | COSM28026 | c.1621A>C | p.Met541Leu | 0.11 | 1516 | 170 |
| KIT | COSM1325 | c.2586G>C | p.Leu826= | 0.11 | 1546 | 168 |
